# The Unique Role of Cutting Balloon Angioplasty in Debulking Multiple Nodular Calcium in Osteoproximal and Mid-left Anterior Descending Coronary Artery

**DOI:** 10.7759/cureus.59740

**Published:** 2024-05-06

**Authors:** Dibyasundar Mahanta, Saran Mohanan, Pranjit Deb, Debasis Acharya, Debasish Das

**Affiliations:** 1 Cardiology, SUM Hospital, Bhubaneswar, IND; 2 Cardiology, All India Institute of Medical Sciences, Bhubaneswar, Bhubaneswar, IND

**Keywords:** left anterior descending coronary artery, mid, osteoproximal, nodular calcium, cutting balloon

## Abstract

We report a rare case of multiple nodular calcium in the left anterior descending coronary artery in an octogenarian presenting with unstable angina. Dilatation with the noncompliant and scoring balloon could not yield the nodular calcium and it was only the cutting balloon that could yield the nodular calcium and successful coronary angioplasty could be accomplished with good angiographic results with distal Thrombolysis in Myocardial Infarction (III) flow. This case demonstrates the unique role of cutting balloons in the angioplasty of coronary lesions with multiple nodular calcium.

## Introduction

The role of nodular calcium in making the coronary intervention complex is well-known in today’s era of complex and high-risk interventional procedures. Debulking the nodular calcium is the principal strategy in lesion preparation before stenting with drug-eluting stents. Nodular calcium requires therapy either with shaving or cracking. Nodular calcium can also be sliced apart using cutting balloons [1], facilitating further passage of noncompliant balloons and stents. The most important aspect of the cutting balloon is that it slices apart the coronary calcium, creating definite dissection which requires further addressal with a drug-eluting stent. Shaving refers to the use of rotational atherectomy whereas cracking refers to the use of intravascular lithotripsy, noncompliant balloon, or scoring balloon [2,3]. We present a rare case in which the osteoproximal and mid-left anterior descending coronary artery (LAD) had multiple nodular calcium and noncompliant and scoring balloon did not help yield the nodular calcium. It was difficult to pass the stent across the nodular calcium with multiple failed attempts. The cutting balloon only yielded the lesion and sliced the multiple nodular calcium with multiple dissections, after which we were able to negotiate the drug-eluting stent further. There was a good angiographic result with distal Thrombolysis in Myocardial Infarction (TIMI) III flow. The patient could not afford intravascular lithotripsy or rota ablation and the use of a cutting balloon achieved successful coronary angioplasty in the presence of nodular calcium.

## Case presentation

**Figure 1:**
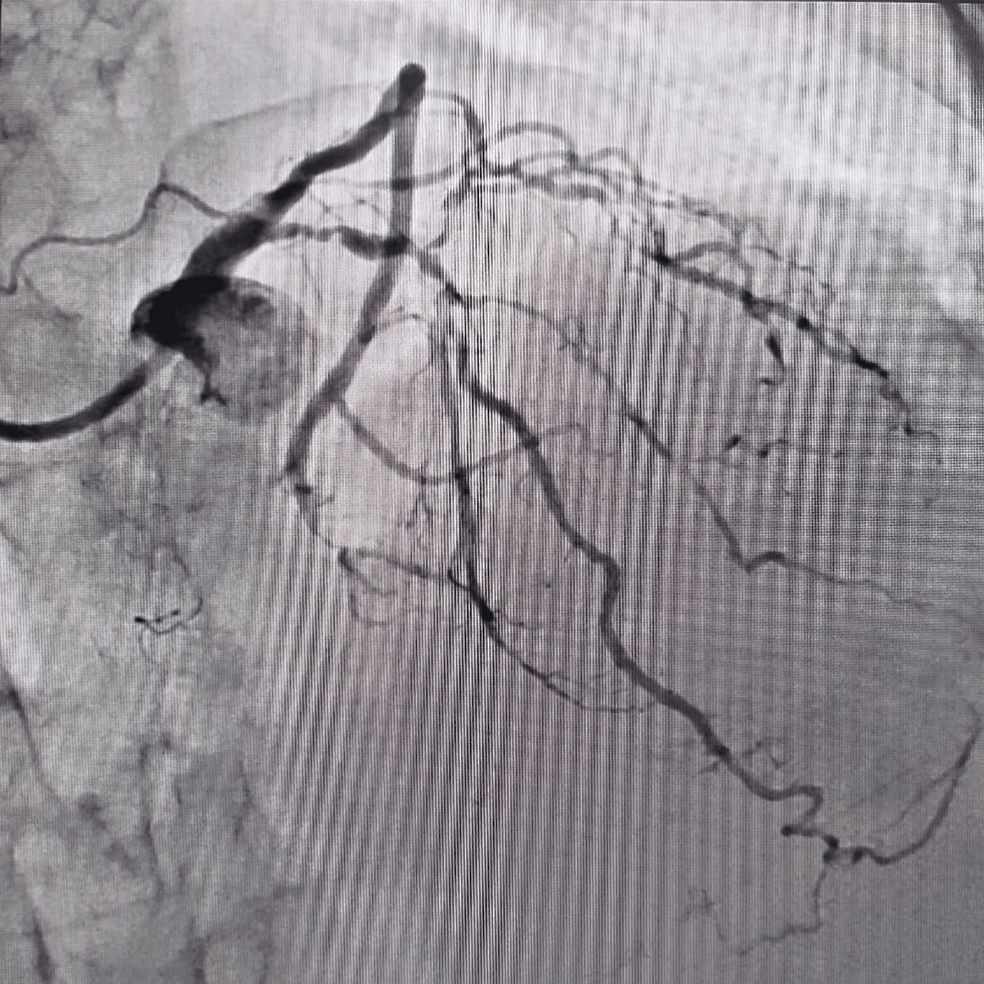
Multiple nodular calcium in the osteoproximal and mid-left anterior descending coronary artery.

An 80-year-old diabetic, hypertensive, and smoker male presented to the cardiology outpatient department with angina in the last three days along with diaphoresis and shortness of breath. During the presentation, his heart rate was 80 beats per minute and his blood pressure was 154/90 mmHg in the right arm supine position. His electrocardiogram revealed downsloping ST depression with T-wave inversion in the anterior precordial leads. His troponin T level was not elevated. His echocardiography revealed regional wall motion abnormality in the LAD with mild left ventricular systolic function (ejection fraction = 45%). He was subjected to a right transradial coronary angiogram which revealed subtotal occlusion in the osteoproximal and mid-LAD with multiple nodular calcium (Figure 1). Given these findings, he was planned for coronary revascularization.

**Figure 2:**
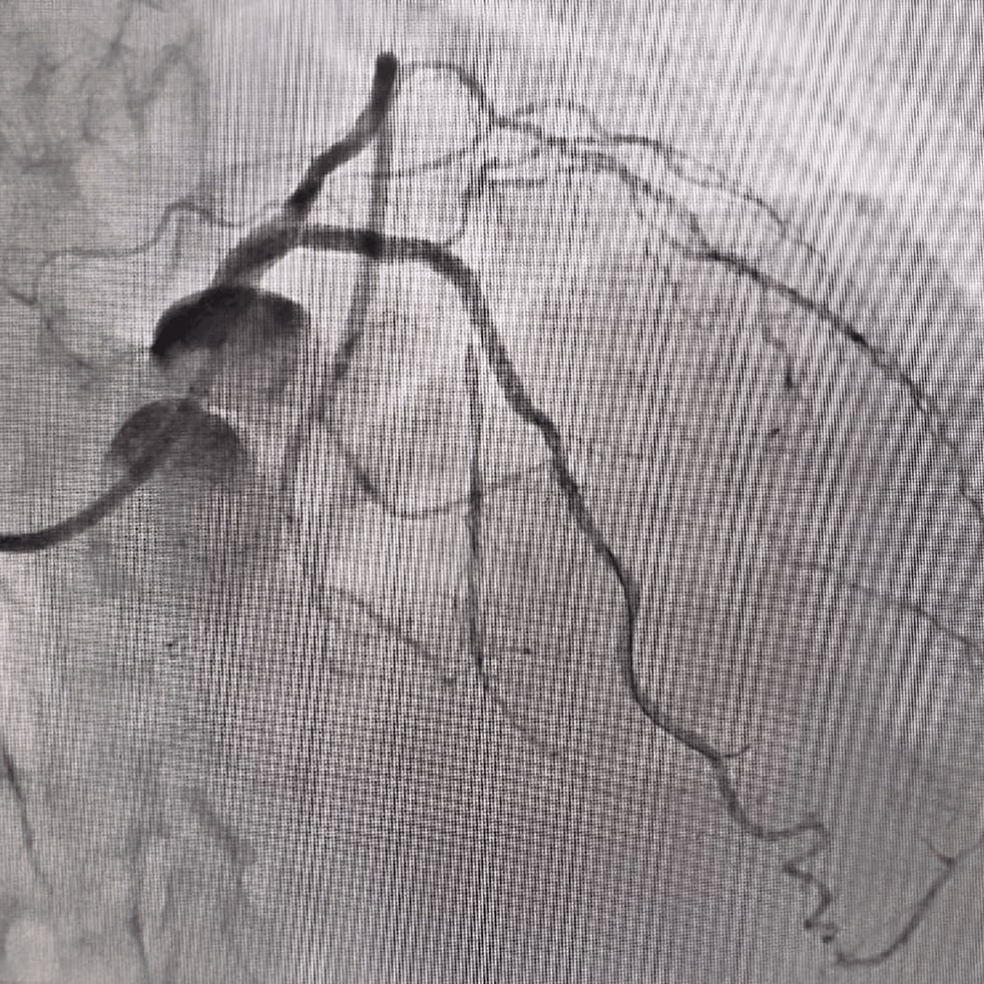
Good angiographic result post-cutting balloon angioplasty.

The left main coronary artery was engaged with Extraback Up 6F 3.5 and a 0.014" Fielder FC guide wire was used to cross the lesion in LAD. The lesion in osteoproximal and mid-LAD was dilated with a 2.5 × 10 mm noncompliant balloon at 18-20 atm pressure but it did not yield the lesion. Subsequently, a scoring balloon measuring 2.5 × 12 mm was used to dilate the lesion which also did not yield the coronary lesion. It was impossible to deploy a 3 × 34 mm drug-eluting stent across the lesion as it could not cross the lesion. Hence, it was decided to prepare the lesion with multiple calcium nodules with a cutting balloon. A 3 × 12 mm cutting balloon was expanded across the lesion from the distal to the proximal segment at 12-14 atm pressure which resulted in multiple cuts and dissections in the coronary artery. Then, it was again dilated with a 3 × 12 mm noncompliant balloon at 16-18 atm pressure after which we were able to negotiate the stent across the lesion. We deployed a 3 × 36 mm drug-eluting stent across the lesion at 14 atm pressure and post-dilated with a 3.5 × 10 mm noncompliant balloon at 18-20 atm pressure. Post-procedure, there was good angiographic result with distal thrombolysis in myocardial infarction (TIMI)III flow (Figure 2), and the patient was hemodynamically stable without any periprocedural complication. Echocardiography immediately after and six hours post-procedure did not reveal any pericardial effusion. The patient was kept on dual antiplatelets, i.e., aspirin and ticagrelor, with a high-dose statin (atorvastatin 80 mg) and an optimum beta-blocker dose. The patient was discharged angina-free without any shortness of breath, and he was doing fairly well on follow-up after three months.

## Discussion

Cutting balloons have rectangular scoring edges that lock the device in place across the lesion. As the cutting balloon is locked in place, less device slippage or melon seedling occurs. The force exerted by a cutting balloon is 15-25 times more than that of a conventional semi-compliant balloon. The helical nitinol element of the cutting balloon creates a uniform luminal arrangement. As a cutting balloon does not slip during inflation, the risk of damage to the healthy endothelium at the edges is much less compared to a semi-compliant or noncompliant balloon. The advantage of cutting balloons is that they rarely cause flow-limiting dissections [1] and require adjunctive stenting. Cutting balloons have a low rate of dissection and minimal perforation. Of interest, the use of cutting balloons is that they slice the nodular calcium into rectangular slabs with minimal dissections and make passage of coronary hardwires including stents and balloons easier. Cutting balloons with three protruding blades produce sharp cuts in the calcium nodules with minimal dissection. Multiple calcium nodules in the osteoproximal and mid-segment of LAD could not be dilated with conventional noncompliant and scoring balloons. With the help of a cutting balloon at 12-14 atm pressure, the lesion yielded and we could negotiate the stent across the lesion. The main disadvantage of cutting balloons in the calcific lesions is that they may not be effective in much deeper calcium as the blades will not reach there compared to intravascular lithotripsy which is safe and effective in deeper calcium. As the patient could not afford intravascular lithotripsy or rota ablation, the last available option was the use of a cutting balloon. Cutting balloons provides scoring marks wherein the phenomenon of plaque shift or snow plow phenomenon is less with cutting balloons compared with semi-compliant or noncompliant balloons. Cut, score, press, shock, and ablate are the five modalities in debulking coronary calcium [2-6] to achieve good angiographic results and clinical outcomes. Our case is an elegant demonstration of the unique role of cutting balloons in the presence of multiple nodular calcium in critical coronary regions such as osteoproximal and mid-LAD. The cutting balloon has rare complications such as tip break, balloon failure, and balloon rupture in severely calcified lesions. In summary, cutting balloons alter the landscape for treating coronary artery disease. Besides calcium nodules, cutting balloons have a promising role in left main, bifurcations, ostial lesions, fibrotic coronary lesions, and hard in-stent restenosis. Cutting balloons can also be called a “poor man scoring balloon” in densely calcified lesions with multiple calcium nodules. 

## Conclusions

We report a rare case of successful debulking of multiple nodular calcium with cutting balloon angioplasty which not only achieved good angiographic results but also distal TIMI III flow in an octogenarian presenting with ongoing angina. Cutting balloon angioplasty helps in calcified lesions in situations with unpassable devices. We suggest possible benefits of cutting balloon angioplasty in the presence of multiple nodular calcium in the coronary arteries when conventional noncompliant and scoring balloon fails. Future studies are warranted to confirm these findings.
